# Eosinophilic myocarditis during treatment with olanzapine - report of two possible cases

**DOI:** 10.1186/s12888-016-0776-y

**Published:** 2016-03-17

**Authors:** Torkel Vang, Mary Rosenzweig, Christina Hedegaard Bruhn, Christoffer Polcwiartek, Jørgen Kim Kanters, Jimmi Nielsen

**Affiliations:** Aalborg University Hospital, Psychiatry, Brandevej 5, 9220 Aalborg, Demmark; Danish Health and Medicines Authority, Copenhagen, Denmark; Department of Clinical Medicine, Aalborg University, Aalborg, Denmark; Laboratory of Experimental Cardiology, University of Copenhagen, Copenhagen, Denmark; Department of Cardiology, Herlev University Hospital, Copenhagen, Denmark

**Keywords:** Olanzapine, Clozapine, Eosinophilic, Myocarditis, ADR, Hypersensitivity

## Abstract

**Background:**

Drug-induced eosinophilic myocarditis is a life-threatening and frequently overlooked condition. The prevalence of myocarditis in clozapine-treated patients may be as high as 3 %. An association between olanzapine and myocarditis has not previously been described, but given the chemical similarity between olanzapine and clozapine, we hypothesized the existence of such an association. We searched the spontaneous adverse drug reports database of the Danish Health and Medicines Authority for olanzapine and myocarditis in the period from October 21, 1996 to – June 03, 2015. We identified two fatal cases of eosinophilic myocarditis associated with the use of olanzapine.

**Case presentation:**

Case 1 was a 39-year-old Caucasian man with known substance abuse and schizophrenia. He was found dead in his home. Olanzapine was prescribed at day -54, and dose at time of death was 40 mg/day. Post-mortem toxicological examination demonstrated presence of olanzapine, morphine, venlafaxine and oxazepam. Syringes indicating substance abuse were found in his home.

Case 2 was a 36-year-old Caucasian man diagnosed with schizophrenia was found dead unexpectedly. There was no history of substance abuse. Current treatment was olanzapine 20 mg/day +5 mg as PRN (prescribed for almost 4 years), aripiprazole 30 mg/day (prescribed for 6 months) and mirtazapine 30 mg/day (prescribed for 6 months).

Both cases of eosinophilic myocarditis were confirmed by autopsy findings and both patients received olanzapine in doses exceeding the recommendations.

**Conclusion:**

Olanzapine may have contributed to and/or worsened the two reported fatal cases of myocarditis. Additional studies are required to establish a causal link between olanzapine and eosinophilic myocarditis.

## Background

Myocarditis, defined as an inflammatory condition of the myocardium, may lead to myocardial fibrosis, arrhythmias and sudden cardiac death. Etiological factors for myocarditis include i) infections (with viruses, bacteria, fungi, protozoa or parasites), ii) autoimmune mechanisms, and iii) substances/drugs (toxic effects or hypersensitivity reactions). The diagnosis is based on endomyocardial biopsies where histological examination reveals inflammatory infiltrates and myocardial necrosis in the same section (the so-called Dallas criteria). Additional analyses such as immune-histochemistry, molecular techniques (e.g. polymerase chain reaction) and myocardial imaging may also be required [1]. Eosinophilic myocarditis (EM), which is a rare form of myocarditis constituting 0.5 % of all myocarditis autopsy cases [2], is characterized by abundant eosinophils in the inflammatory infiltrate. These eosinophils release toxic factors that induce apoptosis and necrosis of myocytes. Often, EM is not recognized in living patients, and the diagnosis is therefore given at the time of autopsy [3]. EM is associated with systemic autoimmune disorders and hypereosinophilic syndromes. Importantly, drug-induced hypersensitivity reactions can also cause EM. The most common agents in this regard are clozapine, antibiotics and antiphlogistics [4, 5]. A recent study from Australia has suggested that clozapine-induced myocarditis is a highly overlooked adverse effect with an actual prevalence as high as 3 % [6].

**Fig. 1:**
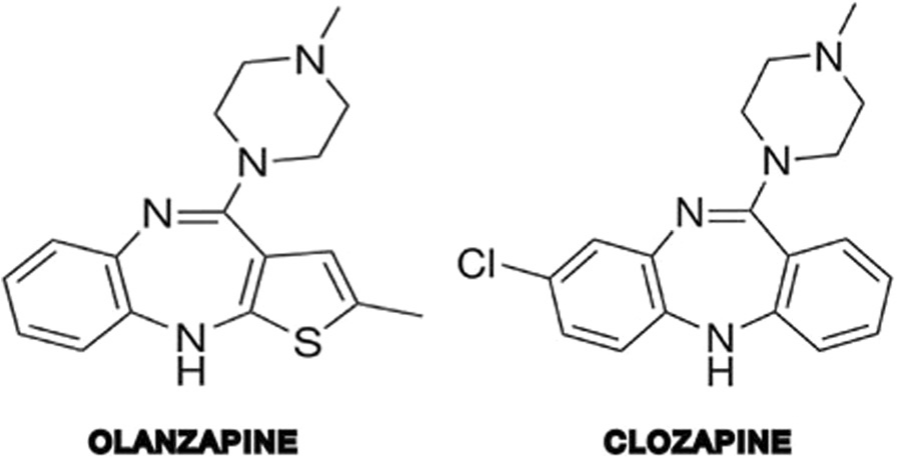
Chemical structure of clozapine and olanzapine

In addition to clozapine, there are anecdotal reports suggesting a possible association between other psychotropic drugs and the development of EM. These include the tricyclic antidepressants amitriptyline [7] and imipramine [8], the first generation antipsychotic (FGA) perphenazine [9], and more recently the second generation antipsychotic (SGA) quetiapine [10]. Furthermore, animal studies have revealed that different antipsychotics to a various extent induce cardiac alterations (ventricular hypertrophy, myocardial necrosis and fibrosis) comparable to what can be seen in myocarditis [11]. Altogether, this suggests that additional antipsychotics may cause EM. As clozapine-induced myocarditis may be overlooked, we proposed that this might also be the case for olanzapine, which is chemically related to clozapine as shown in Fig. 1.

## Method

We searched the spontaneous adverse drug reports database of the Danish Health and Medicines Authority for olanzapine and myocarditis in the period from October 21, 1996 to – June 03,.2015. In total we identified 405 adverse drug reaction (ADR) reports involving olanzapine, including, two fatal cases of EM associated with the use of olanzapine. No non-fatal cases of olanzapine associated myocarditis were found. Case 2 has been published elsewhere with the focus on sudden cardiac death and association to aripiprazole [12]. Presentation of cases was based on ADR reports with no direct access to patient records.

## Case presentation

### Case 1

Thirty-nine-year-old man with known substance abuse and schizophrenia was found dead (day 0). Syringes with traces of morphine and tetrahydrocannabinol were found close to the body. There was no reports on previous physical disease. Olanzapine was prescribed at day -54, and dose at time of death was 40 mg/day. At day -48 pregabalin was prescribed for anxiety. Venlafaxine was prescribed for major depression; dosage was not specified. Additionally, oxazepam was prescribed for anxiety and used for various dosages for up to 5 years. Post-mortem toxicological examination demonstrated presence of olanzapine (0.15 mg/kg), morphine (0.12 mg/kg), venlafaxine (0.22 mg/kg), oxazepam (0.07 mg/kg), diazepam (0.31 mg/kg) and pregabalin (3.1 mg/kg). Plasma levels were not reported but described as within the therapeutic range. At autopsy, cause of death was considered to be myocarditis, which probably had led to a lethal arrhythmia. Upon histological examination of cardiac tissue, there were several sections with myocardial necrosis and abundant eosinophilic granulocytes, indicating EM.

### Case 2

Thirty-six-year-old man diagnosed with schizophrenia was found dead (day 0). The death was unexpected as the patient had no substance abuse, suicidal ideation or known physical disease. Current treatment was olanzapine 20 mg/day +5 mg as PRN (prescribed for almost 4 years), aripiprazole 30 mg/day (prescribed for 6 months) and mirtazapine 30 mg/day (prescribed for 6 months). Post-mortem toxicological analyses revealed aripiprazole 0.7 mg/kg, mirtazapine 0.07 mg/kg and olanzapine 0.10 mg/kg. Autopsy demonstrated fibrous tissue in the inferior part of the cardiac septum. Histological examination of the fibrous area in the septum as well as from macroscopically normal structures in the heart, showed presence of interstitial fibrosis and myocyte necrosis. These lesions were of different age, and the more recent ones contained inflammatory cells with a dominance of eosinophilic granulocytes. Certain cause of death could not be established, but cardiac arrhythmia after EM was suspected.

## Discussion

Olanzapine, which has a chemical structure relatively similar to that of clozapine, has so far not been linked to myocarditis. Here, we report two cases where post mortem analyses indicated death due to EM. For both cases, olanzapine may have contributed to and/or worsened the disease process.

In general, EM is a rare disease constituting only 0.5 % of all cases of myocarditis. Since the symptoms may be vague/nonspecific or self-limiting and the clinicians tend to miss the diagnosis, the condition is often first identified at the time of autopsy. This was also the case for our two patients and may also explain why no cases of non-fatal EM were found in the database.

Notably, drug-induced hypersensitivity reactions and/or toxic effects are important causes of EM. Given the paucity of autoimmune manifestations, hypereosinofilic syndromes and parasitic infections in our two patients, it is most likely that they died from drug-induced EM.

Toxicological analyses of blood from case 1 indicated presence of olanzapine, venlafaxine, morphine, pregabalin, diazepam and oxazepam, none of which in abnormal levels. Neither of these have previously been associated with EM. For the following three reasons, we favor a model where EM was induced by olanzapine in case 1. First, olanzapine is structurally related to clozapine, which is well-known to cause EM. Furthermore, other psychotropics such as the SGA quetiapine and the tricyclic antidepressants amitriptyline and imipramine, have been linked to myocarditis, and they are all chemically related to clozapine. Second, while the patient had used the other medicines for several months/years, olanzapine was introduced 8 weeks before the time of death. Noteworthy, clozapine-induced EM most often becomes clinically relevant after 2 to 4 weeks of treatment [13]. And third, animal studies indicate that several antipsychotics can cause various cardiac abnormalities reminiscent of myocarditis, suggesting that there might be a class effect (Belhani et al. [11]).

Toxicological analyses of blood from case 2 revealed presence of aripiprazole, olanzapine and mirtazapine. Importantly, the concentration of aripiprazole was significantly elevated. While this patient had taken olanzapine for almost 4 years, treatment with aripiprazole and mirtazapine was initiated about 6 months prior to the time of death. Based on this information, it is tempting to suggest that EM was induced by aripiprazole in this patient. However, a contributing role by olanzapine is still a possibility, either on its own or in combination with aripiprazole. Noteworthy, histological examination of the heart revealed several areas with fibrosis and myocyte necrosis. Since these lesions were of different age, there is a possibility that some of them were more than 6 months old, e.g. from the period when the patient was treated with olanzapine as monotherapy. The subsequent introduction of aripiprazole may then have worsened the condition, for instance as a consequence of the class effect of antipsychotics described above. Furthermore, an involvement of mirtazapine can not be excluded, although it has never been linked to myocarditis in the past.

Olanzapine-induced EM may be less common because of lower therapeutic dosage than clozapine. Although not confirmed by solid evidence, inventories have suggested that drug hypersensitivity reactions may be more common in less potent drugs, i.e. therapeutic dosages >100 mg/day, most likely because of higher production of reactive metabolites [14]. In both cases, olanzapine was used at higher dosages than recommended (20 mg as stated in the summary of product characteristics), which may have contributed to the development of EM. Lower production of reactive metabolites with more potent drugs, i.e. olanzapine, may warrant longer time to achieve sufficient levels of reactive metabolites. This is also supported by the fact that EM developed later than the usual 2–4 weeks observed with clozapine.

In general, when drug-induced EM is diagnosed, the offending drug should be removed immediately. In critically ill patients with cardiac failure, hemodynamic stability must be ensured using the proper means. Otherwise, standard medical treatment for cardiac failure should be given. Treatment with immunosupressants is warranted, usually corticosteroids, but other agents have also been used successfully (e.g. azathioprine, mycophenolate) [1].

The data presented in this paper should be interpreted within their limitations, e.g. both patients were prescribed other medications and our limited information about the two fatal cases.

## Conclusion

Here, we report a possible association between olanzapine and EM. However, there is a paucity in well-controlled molecular, cellular and whole-system experiments establishing a causal explanation. More studies are clearly warranted to elaborate on this topic. In the mean time psychiatrists should be aware of myocarditis when patients developing fever and flu-like symptoms.

## Consent

Informed consent was not warranted as this case series only reports data from the Danish Health and Medicines Authority adverse drug reports database with no direct contact to patients or relatives. Danish Health and Medicines Authority have approved the publication of the cases.

## Abbreviations

ADR: adverse drug reaction
EM: eosinophilic myocarditis
FGA: first generation antipsychotics
PRN: *pro re nata*
SGA: second generation antipsychotics
